# Allergic contact dermatitis to slime^☆^^☆☆^

**DOI:** 10.1016/j.abd.2019.06.008

**Published:** 2020-02-15

**Authors:** Nabila Scabine Pessotti, Mariana de Figueiredo Silva Hafner, Mellanie Starck Possa, Rosana Lazzarini

**Affiliations:** a Skin Allergy Sector, Department of Medicine, Dermatology Clinic, Santa Casa de Misericórdia de São Paulo, São Paulo, SP, Brazil; b Improvement Course of Skin Allergies, Dermatology Clinic, Santa Casa de Misericórdia de São Paulo, São Paulo, SP, Brazil

Dear Editor,

Slime is the colloquial name of a viscoelastic product used as a toy that has gained popularity in recent years among children and adolescents worldwide. It may be home-made or industrialized. The former is obtained by mixing cosmetic and household products, including shaving foams, contact lens solutions containing boric acid, glues, liquid detergents and powdered soap, among others.^1^

We report the case of a 9-year-old girl, with personal history of atopy for 5 years with periods of exacerbation related to slime manipulation. The patient's mother informed that she used to play with both: a commercial slime bought at a toy store, and also a home-made one, created by herself using body moisturizer, boricated water, glue, baking soda, blue food dye and shaving foam.

Dermatological examination showed intense palmar erythema, with multiple vesicles associated with desquamation and some exulcerated areas (Fig. 1). Treatment was introduced with 0.5 mg/kg/day systemic corticosteroid therapy and, after 2 months, patch test was performed using the Brazilian standard series, and also complementary aqueous solution of methylisothiazolinone (0.02%) and a part of the industrialized slime (even knowing the possibility of false-positive reactions because of the potential irritants in its composition) (Fig. 2). The 96 h-reading showed positive results for Kathon CG 2 + (a mixture of methylisothiazolinone and methylchlorothiazolinone), and also for isolated methylisothiazolinone (MI) 2 + (Fig. 3), which is a component present in the specific moisturizer, glue and shaving foam used by the patient in manufacturing the homemade slime. The company responsible for manufacturing the industrialized slime was contacted, and they informed that the only preservatives used in its composition were the parabens, which had negative results in the patch test.

**Figure 1:**
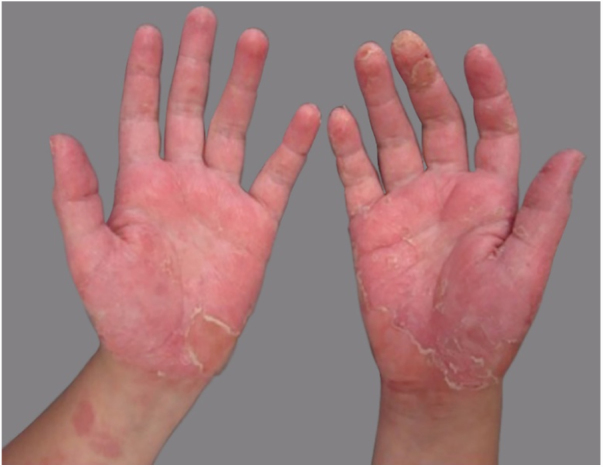
Erythema, scaling and vesicles on palmar surface.

**Figure 2:**
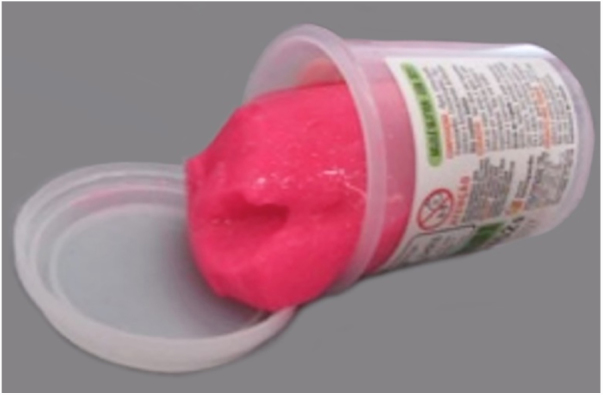
Aspect of industrialized product.

**Figure 3:**
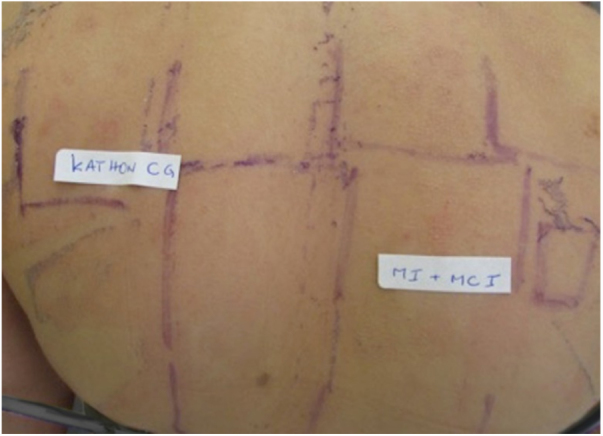
Positive patch test to Kathon CG (++).

Slime can cause both Irritant (ICD) and Allergic Contact Dermatitis (ACD), especially when it is home-made, and so is prepared with the addition of different commercial products. When irritant, boric acid, alcohols and glycol compounds are responsible for the aggression of the skin barrier.^2^ On the other hand, allergens such as methylisothiazolinone and methylchlorothiazolinone are well recognized as causing ACD, and are used as preservatives in industrialized and cosmetic products, such as moist wipes, moisturizers, makeup cleaners glues, among others.^3^ In the present case, this preservative was present in the body moisturizer, the shaving foam and the glue used by the patient, revealing the importance of identifying the allergens in the numerous products that may be used.

Atopic patients are prone to the development of ACD due to deficiency of the skin barrier and also to frequent use of skin care products such as body moisturizers containing possible allergens. In the case reported, the patient had hand dermatitis related to atopy, with exacerbation after slime manipulation, resulting in ACD.

In the last year, several reports of ICD and ACD caused by slime were published, what should alert parents and health professionals to the risks of this play, especially when homemade.

Anderson et al.^4^ analyzed the ingredients of 48 slime recipes available on the internet and demonstrated that the most common were fragrance mix I and II, and preservatives such as propylene glycol and MI (the positive allergen in our patient's patch test).^4^

Clinically, the causes of hand dermatitis may be indistinguishable. Patch test is essential, not only for differential diagnosis (ICD and ACD), but also to detect the causative allergen. It is important to identify it, as these substances are also part of patient's everyday products, such as shampoos, soaps and creams. Thus, if there is no precise diagnosis, dermatitis can evolve with relapses, increasing morbidity and making it difficult to control the disease.

## Financial support

None declared.

## Authors’ contributions

Nabila Scabine Pessotti: Elaboration and writing of the manuscript; critical review of the literature.

Mariana de Figueiredo Silva Hafner: Approval of the final version of the manuscript; elaboration and writing of the manuscript; intellectual participation in the propaedeutic and/or therapeutic conduct of the studied cases.

Mellanie Starck Possa: Approval of the final version of the manuscript; elaboration and writing of the manuscript; intellectual participation in the propaedeutic and/or therapeutic conduct of the studied cases.

Rosana Lazzarini: Approval of the final version of the manuscript; conception and planning of the study; effective participation in research orientation; intellectual participation in the propaedeutic and/or therapeutic conduct of the studied cases.

## Conflicts of interest

None declared.

## References

[1] Asher C., Dalan R., Aly M.I. (2018). Home-made Slime”: a novel cause for pediatric burns’ referrals; do we need to raise awareness?. Burns.

[2] Heller E., Murthy A.S., Jen M.V. (2019). A slime of the times: two cases of acute irritant contact dermatitis from homemade Slime. Pediatr Dermatol.

[3] Piazza C.D., Cestari S.C.P. (2018). Contact dermatitis from do-it-yourself slime. An Bras Dermatol.

[4] Anderson L.E., Treat J.R., Brod B.A., Yu J. (2019). “Slime” contact dermatitis: case report and review of relevant allergens. Pediatr Dermatol.

